# Enhancing hand hygiene compliance in healthcare settings: a long time intervention study

**DOI:** 10.3389/fpubh.2025.1588336

**Published:** 2025-08-20

**Authors:** Jingjing Yue, Huijuan Pan

**Affiliations:** ^1^Department of Hospital Infection Management, Xiaodian District People’s Hospital, Taiyuan, China; ^2^Management Office, Jiangsu Provincial Geriatric Hospital (Jiangsu Province Official Hospital), Nanjing, China

**Keywords:** hand hygiene, healthcare workers, hospital infections, PDCA cycle, compliance, intervention effectiveness

## Abstract

**Objective:**

This study aimed to enhance hand hygiene compliance among healthcare workers (HCWs) to reduce the incidence of hospital-acquired infections (HAIs) by employing the Plan-Do-Check-Act (PDCA) cycle, a quality management approach introduced by W. Edwards Deming.

**Method:**

A tailored Hand Hygiene Survey Form was developed based on the Hand Hygiene Technical Specification for Healthcare Personnel and WHO guidelines. Data was collected from January 2017 to December 2023 and Jiangsu Provincial Geriatric Hospital (Jiangsu province official hospital), including hand hygiene compliance metrics (assessed via observations of WHO’s Five Moments for Hand Hygiene), hospital infection cases, and consumption of hand hygiene consumables. A questionnaire survey identified factors affecting compliance, leading to the implementation of targeted interventions, including regular training, performance assessments, promotional campaigns, and monitoring of consumable usage.

**Results:**

The survey revealed that the need for a diverse range of hand sanitizers (95.53%), timely replenishment of consumables (63.70%), and skin irritation from frequent use (48.83%) significantly impact hand hygiene compliance. From 2017 to 2023, there was a significant increase in hand hygiene compliance rate from 49.25 to 86.67%, accuracy rate from 13.02 to 86.67%, and awareness rate from 61.61 to 96.52%. The total consumption of hand sanitizers increased from 6,277,457 mL in 2017 to 18,130,112 mL in 2023, and the daily consumption per bed-day rose from 8.15 mL to 16.65 mL. The hospital infection rate decreased from 2.63% in 2017 to 0.90% in 2023. A strong negative correlation was observed between hand hygiene compliance rate (*r* = −0.962, *p* < 0.001) and hospital infection rates, indicating that higher compliance is associated with lower infection rates.

**Conclusion:**

The continuous application of the PDCA cycle and targeted interventions significantly improved hand hygiene compliance and reduced HAIs. The study emphasized the importance of ongoing monitoring, feedback, and corrective actions. It also highlighted the need for improving the supply and quality of hand hygiene consumables, enhancing education and supervision, and establishing incentive mechanisms to promote hand hygiene compliance. Despite limitations such as potential overestimation of actual hand hygiene consumables usage, the use of intelligent dispensers with identity recognition is recommended for more accurate data capture in future efforts.

## 1 Introduction

In the realm of hospital-acquired infections (HAIs), hand hygiene emerges as a paramount yet underutilized strategy for prevention, particularly for diseases transmitted through contact (1). Despite its proven efficacy, cost-effectiveness, and simplicity, compliance rates among healthcare workers (HCWs) remain disappointingly low in practical settings (2). This discrepancy between potential and practice underscores a critical need for intervention.

The Plan-Do-Check-Act (PDCA) cycle, a quality management approach introduced by W. Edwards Deming, offers a structured methodology to enhance process quality through iterative planning, execution, evaluation, and adjustment (3). This cyclical process is designed to refine and elevate work quality continuously.

HAIs impose a substantial burden on patients, causing significant physical and psychological distress, as well as economic hardship, while also adversely impacting healthcare quality and safety (4). Hand hygiene stands as the most straightforward and economical method for preventing and controlling HAIs, with the potential to reduce infection rates by 20 to 40% (5). The hands of healthcare workers are identified as the primary conduit for the spread of HAIs in significant outbreaks both domestically and internationally (6). Although hand hygiene is a simple practice, improving its adherence has been a persistent challenge for hospital administrators and a focal point for research (7). In clinical practice, low compliance with hand hygiene among HCWs is attributed to various factors, including heavy workloads, insufficient hand hygiene facilities or supplies, and a lack of knowledge about its importance (8).

While prior studies have established the PDCA cycle’s utility in healthcare quality improvement, its application to hand hygiene compliance in resource-constrained settings remains underexplored. Building on Deming’s framework, this study employs a mixed-methods approach to elucidate context-specific determinants of non-compliance at Taiyuan Xiaodian District People’s Hospital. By identifying modifiable factors, we aim to design targeted bundle interventions integrated within a PDCA framework. This research seeks to: (1) Demonstrate the feasibility of PDCA-driven hand hygiene improvement in a Chinese hospital setting; (2) Quantify the impact of sustained interventions on compliance metrics and HAI incidence; (3) Contribute to the global evidence base on scalable quality improvement strategies for hand hygiene.

This study addresses a critical unmet need in infection prevention by operationalizing a theoretically robust, pragmatically implementable model to bridge the evidence-practice gap in hand hygiene compliance.

## 2 Methods

### 2.1 Data sources

This study was carried out at Jiangsu Provincial Geriatric Hospital (Jiangsu province official hospital). Adhering to the “Hand Hygiene Technical Specification for Healthcare Personnel” WS/T313-2009 (9) and the World Health Organization’s (WHO) “Hand Hygiene Compliance Survey Form,” we developed a tailored “Hand Hygiene Survey Form” for our institution. Full-time infection control personnel conducted weekly random inspections of hand hygiene practices across various departments and assessed the knowledge of staff members. Hospital infection case numbers and the concurrent number of inpatients were obtained through the hospital infection management system, in accordance with the “Hospital Infection Monitoring Specification” WS/T312-2009 (10) to statistically analyze the incidence of hospital infections. The diagnosis of hospital infection cases was made based on the “Hospital Infection Diagnostic Criteria (Trial)” issued by the Ministry of Health of the People’s Republic of China in 2001 (11). The consumption of hand hygiene consumables was calculated based on the number of items issued from the hand hygiene consumables warehouse and the number of inpatient bed-days during the same period. The period from January to December 2017 was designated as the pre-intervention phase, and from January 2018 to December 2023 as the post-intervention phase with dynamic interventions.

### 2.2 PCDA methods

#### 2.2.1 Planning phase (P)

To understand the reasons for the low compliance or reluctance of healthcare personnel to perform hand hygiene, the hospital infection control office, through brainstorming sessions, designed the “Hand Hygiene Knowledge and Influencing Factors Survey” and published it on the online platform Wenjuanxing.^1^ From December 1 to 10, 2017, all hospital staff were encouraged to complete the survey. After verifying the completeness of the responses, data was statistically analyzed to identify the main factors affecting hand hygiene compliance among healthcare personnel and to propose and implement targeted bundled interventions.

#### 2.2.2 Implementation phase (D)

Starting from January 2018, bundled intervention measures were implemented with continuous quality improvement within the PDCA cycle.

In line with the WHO’s “Guidelines on Hand Hygiene in Health Care” released in 2009 (12), and the newly issued “Hand Hygiene Technical Specification for Healthcare Personnel” WS/T313-2019 and “General Requirements for Hand Disinfectants” in 2020 by the National Health Commission of China (13), we continuously refined our institution’s hand hygiene procedures, promotional materials, and other related elements.

We posted hand hygiene reminder signs and ensured a timely replenishment of hand hygiene products. Non-touch faucets and hand-drying equipment were installed in all clinical areas, and rapid hand disinfectants were provided in treatment carts and wards. Hand hygiene moments charts were posted on sterile cabinets and treatment carts, handwashing step charts next to sinks, and hand hygiene reminder signs in various areas of the wards. We introduced non-sticky water-based rapid hand disinfectants and offered a variety of types and components of hand hygiene products to clinical departments to enhance compliance.

On May 5th each year, WHO Hand Hygiene Day, we organized related promotional activities, inviting the hospital president, department heads, and nursing supervisors to participate, thereby raising overall hand hygiene awareness. We also promoted hand hygiene knowledge through various forms and channels, such as inviting experts to give lectures, routine training, outpatient hand hygiene education, creating original handwashing dances, holding photography and cartoon contests, and distributing hand hygiene promotional materials. We produced instructional videos on the six-step handwashing method and surgical hand disinfection, which were made available on the hospital’s internal OA system for various departments to view and learn from. In collaboration with the Dongguan Health Bureau, we produced a “Say No to Secondhand” hand hygiene educational film, which was played on hospital multimedia platforms, various public accounts, and the city’s public transportation system. The hospital infection control office conducted periodic hand hygiene training and assessment for healthcare personnel, support staff, new graduates, residents, interns, and visiting scholars. Weekly random assessments were conducted, and at least quarterly comprehensive infection control knowledge assessments were carried out using the Wenjuanxing platform, including hand hygiene knowledge. Each department organized at least two hand hygiene knowledge training sessions per year, and infection control team members monthly checked the hand hygiene knowledge of departmental staff.

#### 2.2.3 Checking phase (C)

The hospital infection control office quarterly monitored the consumption of hand hygiene consumables per bed-day in each department. Target values for hand hygiene consumables consumption per bed-day were set based on the risk level of different departments. The top 10 departments with the largest deviation from the target values were announced on the hospital’s internal OA system, and these departments were required to submit an analysis and corrective feedback form.

Using the WHO “Hand Hygiene Self-Assessment Framework “(HHSAF) (14) for supervision (from January 2023, the Infection Control Workspace was used), the hospital infection control office conducted at least 200 person-times of hand hygiene compliance observations per month, and each department conducted at least 40 person-times per month. Trained infection control personnel used standardized observation forms to record hand hygiene opportunities, compliance status, and technical accuracy, in accordance with the WHO’s “Five Moments for Hand Hygiene” framework (WHO-recommended “Five Moments”: pre-patient contact, pre-aseptic procedure, post-body fluid exposure, post-patient contact, and post-contact with patient surroundings). Data was compared and publicized to enhance department heads’ focus on hand hygiene management and prevent data falsification.

Each year, 10 hand hygiene outstanding departments were selected, rewarded with rapid hand disinfectants and handwashing liquids; hand hygiene data served as an important indicator for the semi-annual evaluation of outstanding infection control departments; hand hygiene indicators were linked to departmental performance to encourage department heads to focus on hand hygiene management.

#### 2.2.4 Acting phase (A)

**Figure 1:**
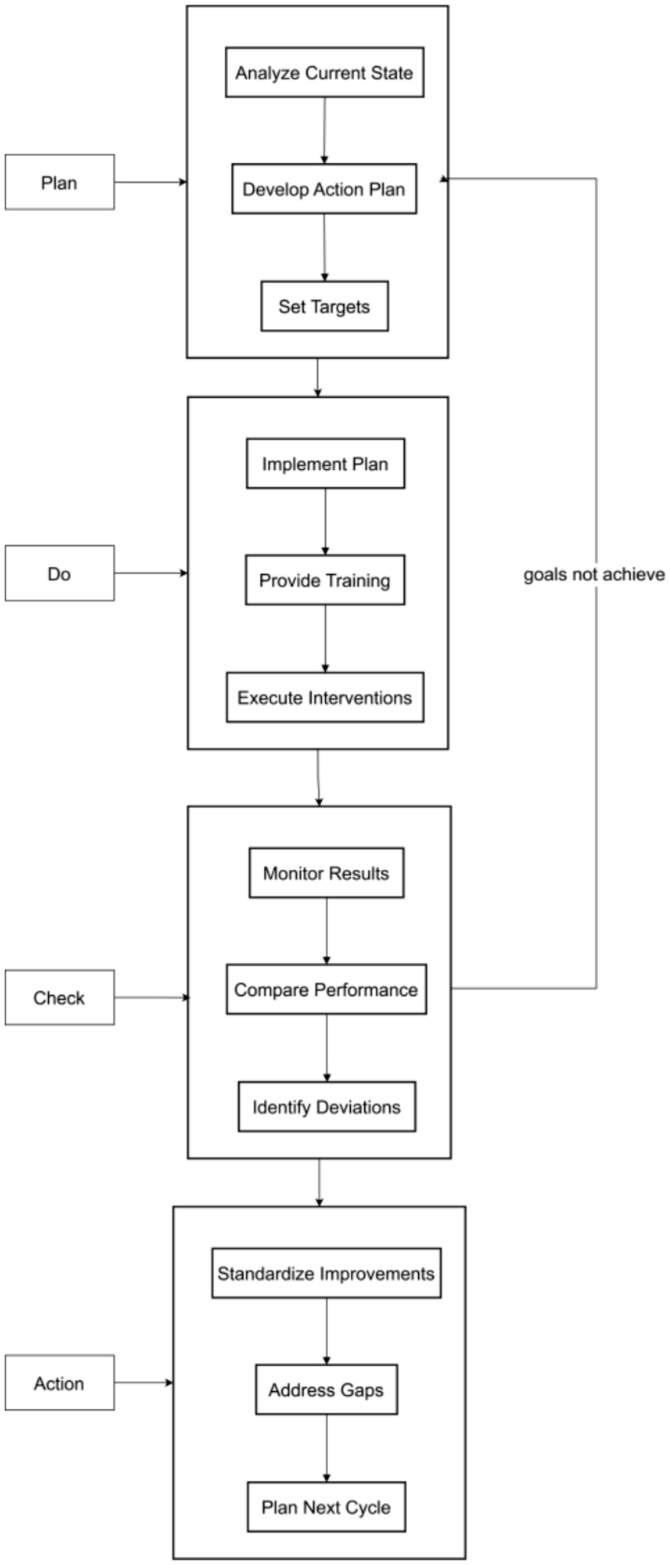
Flow chart of the PDCA cycle.

With an annual cycle, the hospital infection control office consolidated and provided feedback on hand hygiene supervision findings (including compliance rates, technical accuracy, and consumable usage) and relevant data to each department. Departments were required to conduct systematic self-inspections led by departmental infection control teams (comprising department heads, head nurses, and designated infection control officers) using standardized checklists aligned with WHO’s Five Moments for Hand Hygiene framework. Departments, in conjunction with self-inspection, timely summarized and analyzed existing problems and rectification effects, transferring unresolved issues into the next PDCA cycle to gradually improve hand hygiene management quality (see Figure 1).

### 2.3 Hand hygiene consumables and observation procedures

The hand hygiene formulations used in this study primarily included gel-based disinfectants and liquid soaps with active ingredients of ethanol, quaternary ammonium salts, and chlorine compounds. The alcohol-based hand rubs (ABHRs) were commercially procured products, comprising ethanol (60–82% v/v) and isopropanol (70% v/v) formulations (3 M™ Avagard™, Medline®, PURELL®, etc.), aligning with WHO-recommended alcohol concentrations (≥60% v/v) for microbial efficacy. Quaternary ammonium salt (e.g., benzalkonium bromide) and chlorine-based products served as alternatives for ethanol-allergic staff, accounting for <5% of total consumption. Liquid soaps were soap-based and reserved for scenarios requiring visible soil removal (e.g., after blood/body fluid contact) or pre-invasive procedures.

ABHR containers were strategically positioned per the WHO “Five Moments for Hand Hygiene” framework, including: wall-mounted dispensers at patient room entrances, bedside care stations, portable units on treatment carts, and nursing stations. Hospital protocols [aligned with WS/T 313–2019 (13)] designated ABHR as the primary method for non-soiled hands (e.g., before/after patient contact, after touching patient surroundings), while hand washing with soap and water was mandated for visibly contaminated hands, surgical hand preparation, or after handling contaminated equipment.

Observation audits were conducted by trained infection control personnel who completed a WHO-accredited hand hygiene observation training program (inter-rater reliability *κ* ≥ 0.85). Auditors used standardized forms to record hand hygiene opportunities (WHO-recommended “Five Moments”: pre-patient contact, pre-aseptic procedure, post-body fluid exposure, post-patient contact, and post-contact with patient surroundings), compliance status (performed/non-performed), method (ABHR/hand washing), and technical accuracy (adherence to the 6-step handwashing technique). Observations were conducted across all shifts, with monthly audits (≥100 observations/month) in the pre-intervention phase (2017) and weekly audits (≥50 observations/week) in the intervention phase (2018–2023). To mitigate the Hawthorne effect, 20% of audits were unannounced, and compliance data were triangulated with consumable usage metrics (mL/bed-day) for validation.

### 2.4 Calculation of monitoring indicators

The composition ratio of factors affecting healthcare personnel’s hand hygiene compliance was calculated as the number of selections for each option divided by the total number of returned questionnaires, multiplied by 100%.

The hand hygiene compliance rate was defined as the proportion of observed hand hygiene executions (based on WHO’s Five Moments) to the total identified hand hygiene opportunities, multiplied by 100%.

The hand hygiene accuracy rate referred to the proportion of correctly performed hand hygiene actions (adherence to 6-step handwashing technique or proper ABHR application) among all executed hand hygiene events, multiplied by 100%.

The hand hygiene qualified rate was calculated as the proportion of hand hygiene facilities (e.g., dispensers, sinks) meeting inspection standards (e.g., functionality, placement) to the total number of facilities inspected, multiplied by 100%.

The hand hygiene awareness rate represented the proportion of examinees correctly answering ≥70% of hand hygiene knowledge questions (e.g., indications, techniques) to the total number of examinees, multiplied by 100%.

The bed-day consumption of rapid hand disinfectants plus handwashing liquids (mL/bed/day) was calculated as the total consumption of rapid hand disinfectants plus handwashing liquids divided by the number of inpatient bed-days during the same period. The bed-day consumption of paper towels (sheets/bed/day) was calculated as the total consumption of paper towels divided by the number of inpatient bed-days during the same period. The hospital infection incidence rate was calculated as the number of hospital infection patient instances divided by the number of concurrent inpatients, multiplied by 100%.

### 2.5 Statistical methods

Data analysis was performed using the statistical software SPSS 26.0. The results of the questionnaire survey were mainly expressed in frequency. The bed-day consumption of hand hygiene consumables and the hospital infection incidence rate were represented by the annual average value. The linear trend over the years was visualized with linear trend lines to depict temporal changes. The correlation between hand hygiene compliance rate, bed-day consumption of hand hygiene consumables, and hospital infection incidence rate was analyzed using Pearson’s correlation coefficient. A difference was considered statistically significant at *p* < 0.05.

## 3 Results

### 3.1 Prevalence of factors influencing hand hygiene compliance

The survey outcomes, detailed in Table 1, reveal the prevalent factors that impact the adherence to hand hygiene protocols among a cohort of 2,506 medical staff. The data underscores the necessity for a more diverse range of rapid hand sanitizers, as indicated by 2,394 respondents (95.53%), suggesting a substantial demand for product variety to enhance compliance. Additionally, the timely replenishment of hand hygiene consumables is a critical issue, with 1,596 respondents (63.70%) reporting insufficient stock, potentially leading to non-adherence due to lack of resources.

**Table 1:** Survey results on factors affecting hand hygiene compliance among 2,506 medical staff.

Factor	Scaled number	Percentage (%)
There is a need to increase the variety of rapid hand sanitizers in the hospital	2,394	95.53
Hand hygiene consumables are not replenished in time after use	1,596	63.70
Frequent use of hand sanitizers can cause hand irritation	1,224	48.83
Too busy with work to have time	1,016	40.55
The current hand sanitizer is too sticky and uncomfortable for the skin	749	29.87
Allergic to ethanol-based rapid hand sanitizers	331	13.20
Extensive use of hand sanitizers increases departmental costs	202	8.06
Allergic to hand soap	111	4.43
Hand hygiene facilities are inconvenient	76	3.03

Skin irritation resulting from frequent hand sanitizer use was cited by 1,224 respondents (48.83%), indicating a need for gentler formulations. The impact of work pressure on hand hygiene practices is evident, with 1,016 respondents (40.55%) indicating a lack of time due to heavy workloads. The discomfort associated with the stickiness of current hand sanitizers was reported by 749 respondents (29.87%), suggesting a preference for more comfortable alternatives.

Allergic reactions to ethanol-based sanitizers affected 331 respondents (13.20%), while cost considerations were noted by 202 respondents (8.06%), reflecting the economic implications of hand hygiene protocols. Allergic reactions to hand soap were reported by 111 respondents (4.43%), and the inconvenience of hand hygiene facilities was mentioned by 76 respondents (3.03%).

### 3.2 Impact of bundle intervention on hand hygiene metrics

The data presented in Table 2 illustrate significant improvements in various hand hygiene metrics, including the qualified rate, compliance rate, accuracy rate, and awareness rate, from 2017 to 2023. The statistical significance of these changes is highlighted by the chi-square (χ^2^) tests and *p*-values, indicating a positive impact of the interventions on hand hygiene practices and knowledge.

**Table 2:** Comparison of hand hygiene compliance and awareness before and after the intervention measures.

Category	2017	2023	*χ* ^2^	*p*
Hand hygiene facilities				
Total inspections	1,005	1,505		
Number of qualified inspections	411	1,498		
Qualified rate (%)	40.85	99.67	624.11	<0.001
Hand hygiene				
Expected executions	805	9,245		
Actual executions	396	8,015	488.18	<0.001
Compliance rate (%)	49.25	86.67		
Correct executions	53	6,951		
Accuracy rate (%)	13.02	86.67	946.01	<0.001
Hand hygiene knowledge				
Number of examinees	487	2,437		
Number of passes	300	2,351		
Awareness rate (%)	61.61	96.52	359.55	<0.001

### 3.3 Impact of hand hygiene interventions on hospital infection rates

This section analyzes the impact of hand hygiene interventions on reducing hospital infection rates from 2017 to 2023. The data indicates a significant improvement in compliance rates, with a rise from 50.03% in 2017 to 87.13% in 2023.

The total number of inspections and actual executions also experienced a notable increase, with total inspections rising from 572 in 2017 to 6,808 in 2023, and actual executions from 287 to 5,871. Regarding hand sanitizer dosage, the total consumption in milliliters increased from 6,277,457 in 2017 to 18,130,112 in 2023, indicating substantial usage. The daily consumption per bed-day also rose from 8.15 mL in 2017 to 16.65 mL in 2023, pointing to a higher frequency of hand sanitizing practices. Paper towel consumption followed a similar upward trend, with the total consumption climbing from 4,515,542 sheets in 2017 to 9,431,900 sheets in 2023. The daily consumption per bed-day also increased from 5.86 sheets in 2017 to 8.80 sheets in 2023.

**Figure 2:**
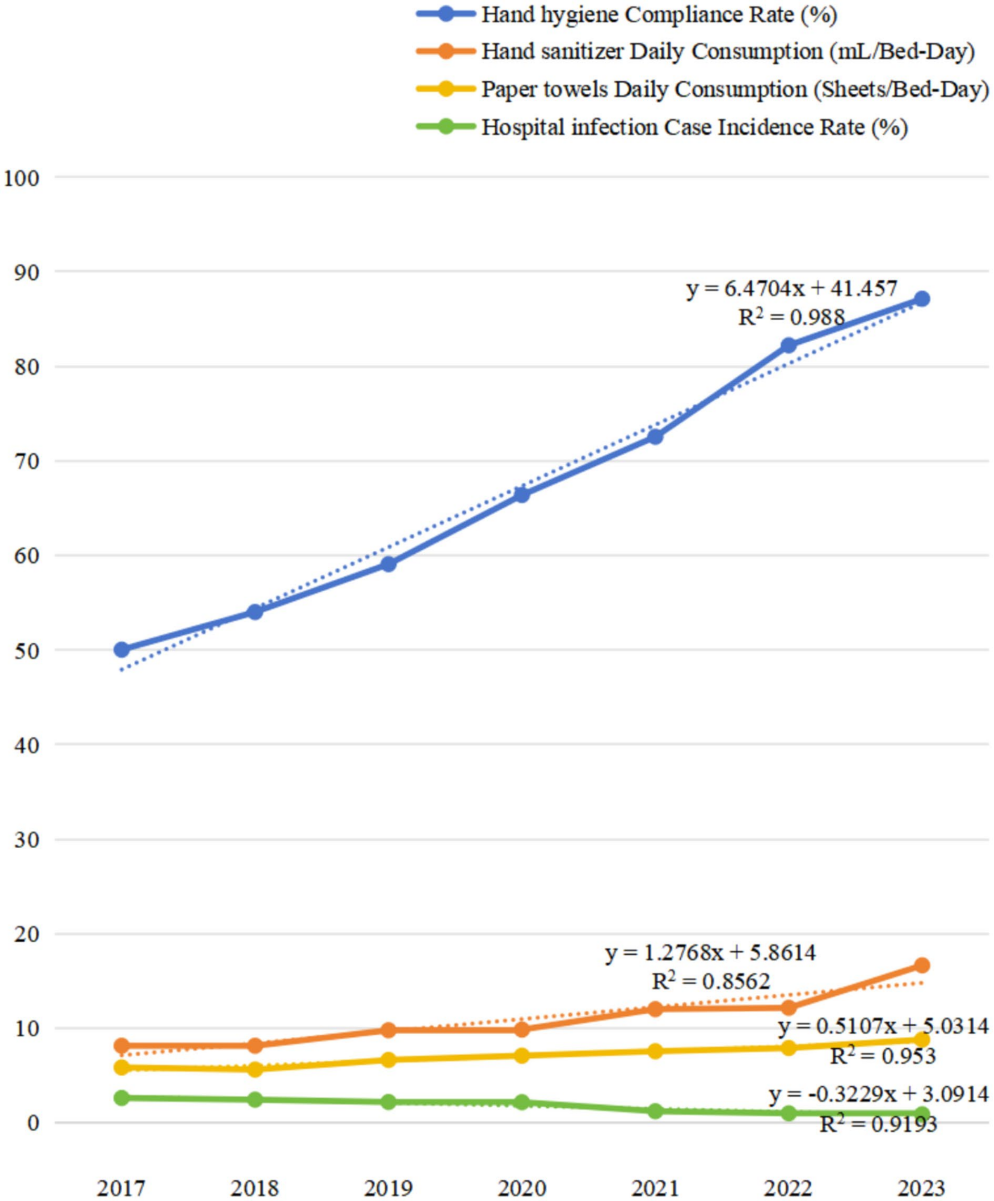
Trends in hand hygiene compliance and hospital infection rates from 2017 to 2023.

Hospital infection rates, measured by the annual number of investigated patient cases and infection cases, generally declined over the years. The annual number of investigated patient cases increased from 99,868 in 2017 to 128,454 in 2023, and infection cases fell from 2,625 to 1,134. This reduction in hospital infections correlates with the improved hand hygiene metrics, suggesting a link between hand hygiene practices and infection control. The case incidence rate also trended downward, from 2.63% in 2017 to 0.90% in 2023 (Table 3; Figure 2).

**Table 3:** Hand hygiene metrics and hospital infection rates from 2017 to 2023.

Category	2017	2018	2019	2020	2021	2022	2023
Hand hygiene							
Total inspections	572	1,089	1,405	1,896	1,603	2,046	6,808
Actual executions	287	583	824	1,260	1,700	1,697	5,871
Compliance rate (%)	50.03	54.01	59.07	66.38	72.54	82.21	87.13
Hand sanitizer dosage							
Total consumption (mL)	6,277,457	6,868,182	7,999,760	12,205,752	15,703,674	14,694,044	18,130,112
Hospitalized patient bed-days	769,896	841,167	821,166	1,239,074	1,319,436	1,210,092	1,090,724
Daily consumption (mL/bed-day)	8.15	8.15	9.79	9.86	12.02	12.16	16.65
Paper towels							
Total consumption (sheets)	4,515,542	4,748,744	5,417,685	8,601,370	9,830,416	9,578,790	9,431,900
Hospitalized patient bed-days	769,896	841,167	821,166	1,239,074	1,319,436	1,210,092	1,090,724
Daily consumption (sheets/bed-day)	5.86	5.63	6.65	7.10	7.57	7.91	8.80
Hospital infection							
Investigated patient cases	99,868	101,996	115,202	98,272	136,645	140,594	128,454
Infection cases	2,625	2,487	2,506	2,133	1,680	1,404	1,134
Case incidence rate (%)	2.63	2.45	2.20	2.18	1.23	1.01	0.90

Table 4 presents the Pearson correlation coefficients between the case incidence of HAIs and hand hygiene compliance rate, as well as the bed-day consumption of hand hygiene products. The data indicates a strong negative correlation between hand hygiene compliance rate and hospital infection rates, suggesting that higher compliance with hand hygiene practices is associated with lower infection rates. The correlation coefficients for hand sanitizer and paper towel consumption also show a negative correlation, indicating that increased consumption of these products is related to lower infection rates.

**Table 4:** Pearson correlation coefficient between case incidence of HAI and HH compliance rate as well as bed-day consumption of HH products consumption.

Variable	*r* (95%CI)	*p*
Hand hygiene compliance rate	−0.962 (−0.997, −0.846)	<0.001
Rapid hand sanitizer + Hand soap bed-day consumption	−0.634 (−0.941, −0.099)	0.033
Paper towels bed-day consumption	−0.926 (−0.974, 0.701)	<0.001

## 4 Discussion

Improving hand hygiene practices among healthcare workers is an ongoing process that requires sustained effort and commitment. Over a 7 years, and Jiangsu Provincial Geriatric Hospital (Jiangsu province official hospital) has implemented a dynamic PDCA cycle, which involves monitoring, feedback, and corrective actions, to enhance hand hygiene compliance. This approach has been instrumental in identifying weak links in management and formulating targeted interventions. By conducting status surveys to understand the current state of hand hygiene practices, implementing bundled measures, and conducting regular inspections and outcome evaluations, the hospital has been able to continuously identify and address issues, thereby improving the quality of hand hygiene practices in a stepwise manner.

The preliminary survey identified key factors affecting hand hygiene compliance among healthcare workers. Targeted interventions, such as introducing non-sticky hand sanitizers and offering a variety of hand sanitizer brands for clinical departments, were implemented. These measures were complemented by multifaceted training, assessment, and promotional activities, which gradually raised awareness of hand hygiene among healthcare workers. Compared to 2017, the hand hygiene compliance rate in 2023 increased from 49.9 to 86.9%, while the daily consumption of hand sanitizers and hand soap, as well as paper towels, increased by 103 and 50.1%, respectively. The incidence rate of hospital infections decreased by 66.79%. A negative correlation was observed between hand hygiene compliance rate, daily consumption of hand sanitizers and paper towels, and the incidence rate of hospital infections, with statistically significant differences, indicating the effectiveness of the bundled interventions.

Notably, 48.83% of respondents reported skin irritation from frequent hand sanitizer use, a finding that may reflect contextual practice gaps rather than inherent product risks. While evidence suggests ABHR generally cause less dermatological irritation than soap and water due to reduced mechanical skin disruption (15), this study’s high prevalence could be attributed to suboptimal application protocols, such as applying ABHR to moist skin after washing (a common error that exacerbates alcohol-induced dryness). Importantly, the study did not specifically evaluate skin irritation associated with hand washing, though existing literature indicates that repeated soap-and-water washing can lead to higher rates of irritant contact dermatitis due to mechanical friction and detergent exposure (15). This disparity highlights a need to differentiate between irritation mechanisms: ABHR-related issues may stem from improper application (e.g., on wet skin), whereas hand washing irritation is more likely linked to prolonged water exposure or harsh soap formulations. Future PDCA cycles should incorporate comparative assessments of skin tolerance between ABHR and hand washing, as well as product-specific irritation profiles (e.g., pH balance, moisturizer content in soaps) to inform targeted interventions. To address this in future iterations of the PDCA cycle, targeted interventions should prioritize hand-drying education, emphasizing the necessity of fully drying hands with paper towels or electric dryers before ABHR application—particularly in scenarios requiring sequential hand washing and disinfection (e.g., handling C. difficile-contaminated materials). Additionally, introducing humectant-enriched ABHR formulations (e.g., glycerin or aloe vera additives) and expanding ethanol-free alternatives (0.5% chlorhexidine gel) could mitigate irritation.

Hospital infections are commonly transmitted through contact, with hands being the primary vector (6). Proper execution of hand hygiene is the most fundamental, simple, and cost-effective method to control the spread of pathogens and reduce the incidence of hospital infections. The CDC’s hand hygiene guidelines emphasize that hand hygiene education and training alone are insufficient and that a combination of other interventions is necessary to improve compliance (15). Without effective supervision, hand hygiene protocols may become mere formalities and fail to be implemented, hence the need to overcome various factors to improve compliance, accuracy, and awareness rates among healthcare workers. Additionally, allergies or poor experiences with hand sanitizers can reduce the willingness of healthcare workers to use them. Providing a variety of hand sanitizers with different formulations, including those free of ethanol but still effective, or those with moisturizing and quick-drying properties, allows healthcare workers to choose based on their experience.

Research has shown that social influence and role modeling are significant factors affecting hand hygiene compliance among healthcare workers (8). Only when every staff member considers hand hygiene as part of their routine responsibilities and clearly understands the moments for hand hygiene can the best measures to interrupt pathogen transmission be taken. By strengthening education and supervision, enhancing awareness and responsibility among healthcare workers, and gradually instilling the habit of hand hygiene as a conscious behavior, the quality of hand hygiene practices can be improved.

The Hawthorne effect, where individuals change their behavior when they know they are being observed, can lead to compliance rates that do not reflect the true situation (16). The daily consumption of hand hygiene consumables is a more objective indicator of the frequency of hand hygiene practices by healthcare workers. Internationally, there is a trend to use the consumption of hand hygiene supplies as an evaluation metric. Additionally, research indicates that hand hygiene consumables are a crucial objective indicator of hand hygiene compliance, and increasing the supply of these consumables can effectively reduce hospital infection rates. With the increase in hand hygiene consumables, the incidence rate of hospital infections gradually decreases, consistent with our findings.

The studies have demonstrated that improving hand hygiene compliance among healthcare workers can reduce hospital infection rates and associated patient costs, shorten patient stays (17). Reducing the average length of stay not only alleviates the burden on patients but also accelerates hospital bed turnover, thereby increasing the economic efficiency and social benefits of the hospital without increasing the number of beds. However, due to long-standing concerns about economic expenditure, there have been instances where clinical departments have not replenished hand hygiene consumables in time after use, leading to low compliance. Additionally, the support from hospital administration is crucial, especially in terms of financial investment, including the renovation and provision of hand hygiene facilities and the cost of consumables.

The studies have shown that providing hand hygiene supplies for free and increasing investment in hand hygiene facilities can improve the compliance and accuracy rates among healthcare workers (18, 19). Therefore, it is recommended that hospitals and departments share the cost of hand hygiene consumables, with a suggestion that well-resourced hospitals could incorporate hand hygiene consumables into departmental cost accounting. At the same time, an incentive mechanism should be established to encourage the practice of hand hygiene, with outstanding departments being rewarded by exemption from all hand hygiene consumables for the year, effectively promoting the compliance of healthcare workers.

The limitations of this study include that hand hygiene consumables were counted based on warehouse issuance records, which may not accurately reflect actual usage due to expired or unused items. Additionally, the study did not systematically assess the impact of the COVID-19 pandemic on hand hygiene practices and hospital infection rates, though the research period (2017–2023) overlapped with the pandemic; such unaccounted external factors (e.g., pandemic-related behavioral changes or surveillance adjustments) may have influenced the observed trends in hand hygiene compliance and HAI incidence, representing a critical gap in causal attribution. Future research should adopt intelligent dispensers with identity recognition to track real-time usage and explicitly model the effects of major public health events on hand hygiene outcomes.

## 5 Conclusion

By applying the PDCA cycle and implementing bundled hand hygiene strategies, strengthening hand hygiene promotion and training, and increasing supervision and management efforts, the hospital achieved continuous improvement in hand hygiene quality, effectively reducing healthcare worker-related hospital infections and fostering a win-win scenario for both the hospital and patients. This approach not only enhanced clinical outcomes but also promoted a spiral upward trend in hospital quality management, shifting from end-of-line quality control to process-oriented continuous improvement.

Notably, during the study period (2017–2023), the hospital infection incidence rate decreased from 2.63 to 0.90%, even amid the COVID-19 pandemic (2020–2022). This decline may be attributed to two synergistic factors: ① Pandemic-related public health measures (e.g., enhanced hand hygiene awareness, mandatory masking) likely complemented the PDCA-driven interventions, accelerating compliance rate growth from 66.38% (2020) to 87.13% (2023); ② The hospital’s adaptive PDCA adjustments, such as integrating pandemic-specific training (e.g., viral transmission precautions) and upgrading hand hygiene facilities, mitigated potential HAI surges despite increased patient workloads. However, the study’s limitation in not systematically modeling pandemic impacts necessitates caution in causal inference. Future research should employ time-series analysis to disentangle the independent effects of PDCA interventions and pandemic measures on HAI dynamics, while exploring whether post-pandemic hand hygiene behaviors sustain long-term improvements.

This work underscores the PDCA cycle’s utility in building resilient hand hygiene systems, highlighting the need for integrated strategies that combine structural improvements (e.g., intelligent dispensers) with behavioral nudges (e.g., real-time feedback) to ensure sustained HAI reduction in both endemic and pandemic contexts.

## Data Availability

The original contributions presented in the study are included in the article/supplementary material, further inquiries can be directed to the corresponding author.

## Footnotes

^1^ https://www.wjx.cn/

## References

[1] Allegranzi B Pittet D. Role of hand hygiene in healthcare-associated infection prevention. J Hosp Infect. (2009) 73:305–15. doi: 10.1016/j.jhin.2009.04.019, PMID: 19720430

[2] Akinsulie OC Aliyu VA Idris I Ajulo S Olukogbe O Ukauwa C. The implications of handwashing and skin hygiene on infectious disease dynamics: the African scenario. Hygiene. (2024) 4:483–99. doi: 10.3390/hygiene4040036

[3] Chen J Li Z Ma W Tang Y Liu C Ma S. Enhancing the timeliness of EMR documentation in resident doctors: the role of PDCA cycle management. BMC Med Educ. (2024) 24:1–10. doi: 10.1186/s12909-024-06134-2, PMID: 39592995 PMC11590573

[4] Gidey K Gidey MT Hailu BY Gebreamlak ZB Niriayo YL. Clinical and economic burden of healthcare-associated infections: a prospective cohort study. PLoS One. (2023) 18:e0282141. doi: 10.1371/journal.pone.0282141, PMID: 36821590 PMC9949640

[5] Clancy C Delungahawatta T Dunne CP. Hand-hygiene-related clinical trials reported between 2014 and 2020: a comprehensive systematic review. J Hosp Infect. (2021) 111:6–26. doi: 10.1016/j.jhin.2021.03.007, PMID: 33744382 PMC9585124

[6] Mouajou V Adams K DeLisle G Quach C. Hand hygiene compliance in the prevention of hospital-acquired infections: a systematic review. J Hosp Infect. (2022) 119:33–48. doi: 10.1016/j.jhin.2021.09.016, PMID: 34582962

[7] McInnes E Phillips R Middleton S Gould D. A qualitative study of senior hospital managers’ views on current and innovative strategies to improve hand hygiene. BMC Infect Dis. (2014) 14:1–12. doi: 10.1186/s12879-014-0611-3, PMID: 25407783 PMC4237732

[8] Alshagrawi S Alhodaithy N. Determinants of hand hygiene compliance among healthcare workers in intensive care units: a qualitative study. BMC Public Health. (2024) 24:2333. doi: 10.1186/s12889-024-19461-2, PMID: 39198830 PMC11351093

[9] Guo L. Implementation of a risk management plan in a hospital operating room. Int J Nurs Sci. (2015) 2:348–54. doi: 10.1016/j.ijnss.2015.10.007

[10] National Health Commission of the People’s Republic of China: Standard for nosocomial infection surveillance in China WS/T312-2009.

[11] Liu JY Wu YH Cai M Zhou CL. Point-prevalence survey of healthcare-associated infections in Beijing, China: a survey and analysis in 2014. J Hosp Infect. (2016) 93:271–9. doi: 10.1016/j.jhin.2016.03.019, PMID: 27140419

[12] World Health Organization. WHO guidelines on hand hygiene in health care. [M]//WHO guidelines on hand hygiene in health care. World Health Organ. (2009). 270 p.

[13] Chen N He W Chen X Li Y Cheng X Liu L. Distribution and characteristics of bacteria on the hand during oropharyngeal swab collection: which handwashing points are affected? J Clin Nurs. (2024) 33:4708–16. doi: 10.1111/jocn.17134, PMID: 38519848 PMC11579574

[14] Sands M Aunger R. Determinants of hand hygiene compliance among nurses in US hospitals: a formative research study. PLoS One. (2020) 15:e0230573. doi: 10.1371/journal.pone.0230573, PMID: 32255783 PMC7138309

[15] Gould DJ Moralejo D Drey N Chudleigh JH Taljaard M. Interventions to improve hand hygiene compliance in patient care. Cochrane Database Syst Rev. (2017) 9:Cd005186. doi: 10.1002/14651858.CD005186.pub4, PMID: 28862335 PMC6483670

[16] Perera A. Hawthorne effect: definition, how it works, and how to avoid it Simply Psychology (2023). Available at: https://www.simplypsychology.org/hawthorne-effect.html

[17] de Kraker MEA Tartari E Tomczyk S Twyman A Francioli LC Cassini A. Implementation of hand hygiene in health-care facilities: results from the WHO hand hygiene self-assessment framework global survey 2019. Lancet Infect Dis. (2022) 22:835–44. doi: 10.1016/S1473-3099(21)00618-6, PMID: 35202600 PMC9132778

[18] Jeanes A Coen PG Drey NS Gould DJ. Moving beyond hand hygiene monitoring as a marker of infection prevention performance: development of a tailored infection control continuous quality improvement tool. Am J Infect Control. (2020) 48:68–76. doi: 10.1016/j.ajic.2019.06.014, PMID: 31358420 PMC7115327

[19] Haenen A de Greeff S Voss A Liefers J Hulscher M Huis A. Hand hygiene compliance and its drivers in long-term care facilities; observations and a survey. Antimicrob Resist Infect Control. (2022) 11:50. doi: 10.1186/s13756-022-01088-w, PMID: 35303941 PMC8931571

